# A qualitative study of tobacco interventions for LGBTQ+ youth and young adults: overarching themes and key learnings

**DOI:** 10.1186/s12889-018-5050-4

**Published:** 2018-01-18

**Authors:** N. Bruce Baskerville, Katy Wong, Alanna Shuh, Aneta Abramowicz, Darly Dash, Aamer Esmail, Ryan Kennedy

**Affiliations:** 1 Propel Centre for Population Health Impact, University of Waterloo, Waterloo, Ontario N2L 3G1 Canada; 2 Sherbourne Health Centre, Toronto, Ontario Canada; 3 Johns Hopkins Bloomberg School of Public Health, Johns Hopkins University, Baltimore, USA

**Keywords:** LGBTQ+, Youth, young adults, cessation, Prevention, Interventions, Focus groups

## Abstract

**Background:**

Smoking prevalence is very high among lesbian, gay, bisexual, transgendered and queer (LGBTQ+) youth and young adults (YYA) compared to non-LGBTQ+ YYA. A knowledge gap exists on culturally appropriate and effective prevention and cessation efforts for members of this diverse community, as limited interventions have been developed with and for this population, and there are very few studies determining the impact of these interventions. This study identifies the most salient elements of LGBTQ+ cessation and prevention interventions from the perspective of LGBTQ+ YYA.

**Methods:**

Three descriptions of interventions tailored for LGBTQ+ YYA (group cessation counselling, social marketing, and a mobile phone app with social media incorporated), were shared with LGBTQ+ YYA via 24 focus groups with 204 participants in Toronto and Ottawa, Canada. Open-ended questions focused on their feelings, likes and dislikes, and concerns about the culturally modified intervention descriptions. Framework analysis was used to identify overarching themes across all three intervention descriptions.

**Results:**

The data revealed eight overarching themes across all three intervention descriptions. Smoking cessation and prevention interventions should have the following key attributes: 1) be LGBTQ+ − specific; 2) be accessible in terms of location, time, availability, and cost; 3) be inclusive, relatable, and highlight diversity; 4) incorporate LGBTQ+ peer support and counselling services; 5) integrate other activities beyond smoking; 6) be positive, motivational, uplifting, and empowering; 7) provide concrete coping mechanisms; and 8) integrate rewards and incentives.

**Conclusions:**

LGBTQ+ YYA focus group participants expressed a desire for an intervention that can incorporate these key elements. The mobile phone app and social media campaign were noted as potential interventions that could include all the essential elements.

**Electronic supplementary material:**

The online version of this article (10.1186/s12889-018-5050-4) contains supplementary material, which is available to authorized users.

## Background

In Ontario, smoking remains a leading cause of cancer with approximately 9800, or 15%, of new cancer cases diagnosed in 2009 attributed to smoking cigarettes [1]. Smoking prevalence is very high among lesbian, gay, bisexual, transgender, and queer (LGBTQ+) youth and young adults (YYA). According to the 2014 Canadian Community Health Survey, 31% of lesbian, gay or bisexual 18–24 year olds from Ontario smoke daily or occasionally compared to 22% of cisgender heterosexual people [2]. For the transgender community in Toronto, 36% report smoking daily and occasionally [3]. Among adolescents of high school age, recent data show that twice as many lesbian, gay, or bisexual (LGB) adolescents report daily cigarette use compared to their non-LGB peers (22% of LGB and 11% of non-LGB) [4]. It is difficult to accurately estimate the size of the LGBTQ+ YYA population as sexual orientation and gender identity are under-reported due to suspicion as to the intended use of the information and the sensitive subject matter [5–7]. However, a Canadian poll revealed 11% of youth (ages 18–34) identify as LGBT and that 5% of Canadians overall identify as LGBT [8].

There are several proposed reasons that explain the high rates of smoking within the LGBTQ+ community, including victimization, discrimination, harassment, internalized homophobia, minority stress, abuse, mental health, direct marketing by the tobacco industry, frequenting bars and nightclubs where smoking may be more normative, other substance use including alcohol, and higher rates of stress, depression, and low socioeconomic status exacerbated by increased homelessness [9–13].

There is a dearth of published research on the effectiveness of tobacco use interventions designed specifically for the LGBTQ+ YYA population [14]. Efforts to reach LGBTQ+ YYA have tended to utilize the same approach to reach each segment of this diverse population, meaning approaches do not tend to differentiate outreach to lesbian, gay, or bisexual people, or individuals who identify as trans or queer. Further, the lack of culturally appropriate LGBTQ+ smoking cessation programs and general health services in communities have been cited as barriers to smoking cessation [15, 16]. When considering tobacco use, the majority of published research has been focused on group cessation counselling (GCC) interventions for the general LGBTQ+ population (e.g., The Last Drag, Queer Quit, Call It Quits, Bitch to Quit, Put It Out, among others) [17–20], and to a lesser extent on broader social marketing campaigns (e.g., Crush, Delicious Lesbian Kisses) [21, 22], or innovative low intensity interventions such as smartphone applications [23]. A review conducted by Lee and colleagues [24] found that group cessation counselling programs for LGBTQ+ persons can be effective but the reach tends to be limited. One social marketing campaign, CRUSH, had media coverage and events targeting LGBTQ+ bar/club-going young adults. The campaign encouraged LGBTQ+ young adult community members to text brand ambassadors in order to receive a text messaging cessation program [21, 25]. Cross-sectional surveys were collected in Las Vegas, Nevada LGBT bars at two time points one year apart and results indicated that 53% of respondents reported exposure to the CRUSH campaign and of those, 86% understood the campaign message [21]. Despite the importance of this research for reaching LGBTQ+ young adult smokers, most programs are targeted to LGBTQ+ older adults and have not undergone rigorous testing to determine their effectiveness as they primarily rely on repeated measures rather than more rigorous controlled designs [14, 16].

A recent systematic review by Berger and Mooney-Somers [16] identified 19 LGBT intervention studies and concluded that LGBT interventions are effective with an overall quit rate of 61% across studies. The review used a study space analysis where studies with high quit rates were differentiated from studies with low quit rates. Intervention studies that incorporated cultural modifications such as meeting in LGBT spaces, LGBT facilitators, and discussion of LGBT specific smoking triggers were more effective than studies that employed strategies such as ex-smoker identity or measuring expired carbon monoxide. The average age of participants across studies was 42 years and interventions specific for LGBT YYA were not identified. For the LGBT community overall, the review concluded that having smoking cessation interventions that are culturally modified may be more effective than interventions designed for the general population [16].

Very few interventions have been designed and evaluated for the LGBTQ+ YYA community, despite the fact that YYA who identify as trans, queer or a sexual minority have significantly higher rates of smoking compared to YYA who identify as cisgender with a heterosexual orientation. The primary purpose of this study was to engage LGBTQ+ YYA to identify the key elements of tobacco use prevention and cessation interventions for their community. This paper explores the necessary elements of prevention and cessation interventions that may impact uptake, use, and ultimately, support behaviour change in tobacco use among LGBTQ+ YYA.

## Methods

### Design and recruitment

We conducted a total of 24 focus groups (FGs) among LGBTQ+ YYA in Toronto, ON (*n* = 18 groups) and Ottawa, ON (*n* = 6 groups) from March to May 2015. Recruitment occurred through purposive and snowball sampling with a variety of methods: posting flyers and verbal announcements in spaces frequented by LGBTQ+ people; Facebook posts on LGBTQ+  friendly group pages; paid Facebook advertisements; e-mails from LGBTQ+ agencies; organizations making calls through social media channels; targeted physical recruitment at bars and nightclubs in Ottawa; and asking participants to refer eligible peers.

Interested participants contacted the project coordinator via e-mail and completed an electronic demographic intake questionnaire. Physical questionnaires were available for those unable to complete the electronic form. Eligible participants were 16–29 years old, a part of the LGBTQ+ community, and a current smoker or recent quitter (defined as having quit in less than six months prior to completing the intake questionnaire). A current smoker was defined as smoking daily or occasionally (less than 7 days per week or less than one cigarette per day). Those who were eligible were assigned to a FG based on their age, city, and LGBTQ+ status. The triaging was done by age and LGBTQ+ status as homogeneity is key to maximizing disclosure among FG participants [26]. On occasion mixed FGs were formed when participant numbers did not allow for a FG of one sexual orientation or gender identity. The project coordinator confirmed attendance and information about FGs via e-mail and attended all sessions. The attendance rate at the FGs was 74% (204 individuals attended out of the 275 who agreed to attend).

### Focus group procedures

Participants were asked to share their opinions, likes and dislikes regarding three hypothetical intervention descriptions: 1) GCC; 2) social marketing (SM) with four potential ad campaign ideas; and 3) mobile phone app (MPA) with social media (see Table 1). The hypothetical intervention descriptions were developed based on findings from a scoping review of the published and grey literature on LGBTQ+ tobacco use prevention and cessation interventions [14], and consultation with community health centres where LGBTQ+ YYA receive services. The same scenarios were used across all the FGs, with the facilitators ensuring that the description of the scenario was appropriate to the LGBTQ+ FG session being held (e.g., the scenario referred to lesbian youth for a FG session with lesbian youth participants).

**Table 1:** Focus Group Materials

Scenarios	Questions asked by facilitator
Group Cessation Counselling	- How do you feel about a group cessation program for people your own age who are LGBTQ, who smoke, and who want to quit smoking? - Can you imagine yourself attending such a program to help you quit smoking? Why or why not? - What are some things that you like about a group program? - What are some things that you don’t like about it?
Imagine that every week you could meet with other LGBTQ youth and young adults who want to quit smoking. A LGBTQ counsellor would run the meetings in a safe and accepting space. The counsellor would share trusted information on how to quit and stay smoke-free, but would leave time for group members to talk about personal experiences with smoking and quitting. Examples of information that might be shared include isolation, loneliness, body image, lifestyle changes, the need for positive support, stressors like the coming-out process, triggers and self-esteem. These groups could be a way to connect with others your age with similar experiences, and promote LGBTQ people supporting LGBTQ people to overcome smoking. The group would also encourage participants to buddy-up with other members so that during the week, people would be able to support each other to stay smoke-free. The group sessions would be 6–8 weeks and have 8–15 people.	
Social Marketing Campaigns	- How do you feel about media campaigns that can help with encouraging quitting or not-smoking? - Of the 4 ideas above, which one did you like the most? Why? - Which one did you like the least? Why? - For the one that you liked the most, is there anything that you would change?
General Population Campaign (A)	
Rather than a campaign that focuses on the LGBTQ community, maybe it’s time to educate the general population about some of the challenges faced by LGBTQ people. Many LGBTQ people experience homophobia, transphobia, heterosexism and are unfairly treated by society. What about a campaign that shows, in first person, some of these hardships? The advertisements would show how challenges such as family stress, peer rejection, victimization, and social anxiety can lead to smoking. For example, in one scenario a young gay male is sitting with his parents and telling them that he is gay; his parents are clearly upset. Another scenario shows a same sex couple going on a date and hearing derogatory comments being murmured and being stared at. After each scene, the individuals are shown reaching for cigarettes to help them cope.	
Tobacco Industry Campaign (B)	
Did you know that tobacco companies zero-in on the LGBTQ community because they think members of the LGBTQ community are an easy target? In fact, one of the biggest tobacco companies created a plan called Project SCUM to manipulate LGBTQ people into buying their cigarettes. Big Tobacco uses manipulative strategies, like sponsoring LGBTQ events and putting glamourized LGBTQ imagery in their advertisements to make it seem like they are allies. The truth is that these tactics are designed to exploit the community.	
Perks of Not Smoking Campaign (C)	
What do you think about a campaign that would show the immediate benefits of not smoking, and the freedom non-smokers feel because they’re smoke-free? For example, ads may show LGBTQ individuals being active. There could be ads that show two young men running, or a young woman lifting weights. The slogan might say “I can run fast and free” or “Nothing can stop me.” Other immediate perks of not smoking that might be part of this campaign could be having more money, sex appeal, and smelling good.	
Awareness and Fact Campaign (D)	
Did you know that for every straight non-trans smoker there are at least 3 LGBTQ smokers and smoking-related illness and death is also much higher for LGBTQ people? Not many people do. What do you think of a campaign that would raise awareness about smoking in the LGBTQ community? This campaign may also feature ads that talk about the challenges that LGBTQ persons have overcome, including smoking. For example, an ad may say: “I overcame the victimization; and the coming out process; I’m not going to let tobacco take me down.”	
Mobile Phone App with Social Media	- How do you feel about a smartphone app and social media campaign for people your own age that are also LGBTQ, who smoke, and who want to quit smoking? - If a smoke-free app was customized to LGBTQ youth and young adults would you use it? Why or why not? - What are some things that you like about a smartphone app and social media program? - What are some things that you don’t like about it?
Do you own a smartphone? Ever play Candy Crush or use Instagram? What if there was an app that could help you quit smoking designed specifically for LGBTQ youth and young adults? For example, this quit smoking app would allow you to create an individualized quit plan where you can set a quit date, it would provide feedback on how you’re doing, record what triggers you to smoke, and give you tips on how to remain smoke-free, as well as links to counselling services. One of the advantages of the app would be access to a peer support network which would connect you to other LGBTQ peers who are also trying to quit or who have already stopped smoking. The app would be part of a bigger social media campaign that would include a webpage, Facebook page, YouTube videos, and Twitter feed with access to more detailed educational resources about smoking and quitting [e.g., nicotine replacement therapy, like gum or the patch]. LGBTQ role models would promote the campaign.	

Facilitators and note takers (one each per location) were trained on the study objectives and the FG protocols (see Additional file 1). Facilitators and note takers all identified as part of the LGBTQ+ community. Participants provided written consent. FGs were conducted in LGBTQ+ community safe spaces. Participants were asked to share their input on three culturally modified intervention descriptions, which were provided to participants one at a time and read aloud by the facilitator. Pen and paper were provided for participants to note any thoughts about the interventions while they were being read or discussed. These hypothetical intervention descriptions were presented to facilitate discussions about general tobacco control prevention and cessation intervention design, and not to refine the three intervention descriptions presented.

The FGs typically contained 9 participants, ranged from 3 to 17 participants, and lasted for 90 min on average. Participants were remunerated with $50 cash. To encourage FG participation, participants who referred an eligible friend to participate in the study were also provided a $5 Starbucks gift card. All FGs were conducted in English and audio-recorded and professionally transcribed along with notes taken from each FG session.

### Data analysis

We analyzed the data using a framework analysis technique [27] that incorporated the stages of familiarization, identification of the framework, indexing, charting, mapping and interpretation. To gain familiarity with the data and develop an initial list of codes, three authors (AS, KW & AA) independently coded the first six FG transcripts and then compared the coding lists for consistency. Any discrepancies in coding were discussed and resolved with the principal investigator (NBB). In this way, each author was able to critically challenge one another on differing perspectives and any potential biases.

After coding of the initial transcripts, the thematic framework containing the a priori issues (i.e., likes, dislikes, and suggestions for improvement) and identified emerging codes was developed and applied to the remaining transcripts by generating major themes and subthemes in relation to the FG questions and categorizing the associated responses iteratively. To maintain the context of FG participant responses, they were listed under the questions from which they were derived and then categorized separately as a type of response. In addition, notes from the sessions were analyzed to help with the interpretation of the transcripts. Throughout the coding process, regular meetings were held between three of the authors (AS, AA, KW) and the principal investigator (NBB) to discuss and refine the thematic framework. Indexing was accomplished by three authors (AS, DD, & AA) by coding each response in NVivo and reliability checked by a fourth author (KW) through review of the NVivo file. At the final stage, the original responses were charted according to the themes and subthemes. Mapping and interpretation involved comparing and contrasting the themes and subthemes to search for final patterns and thematic categories by three authors (AS, DD & KW). Saturation of findings occurred after analysis of the fifteenth FG transcript; however, we conducted an additional nine FGs in an attempt to increase youth and other LGBTQ+ YYA sub-population (e.g., two-spirited) participation. Representative quotes were selected from the FG responses to illustrate key themes.

Member checking was completed with 14 voluntary FG participants to determine the accuracy, credibility and validity of the findings from the qualitative analysis. Member checking took place on November 25, 2015 in Toronto, ON and December 8th, 2015 in Ottawa, ON. After a presentation of the findings from the FG sessions, participants at the member checking sessions provided feedback by addressing: 1) what they thought about the results presented; 2) whether anything was missed; and, 3) whether there was anything else to add. Participants confirmed that the qualitative analysis of FG transcripts captured the key themes. Participants at the member checking sessions were remunerated with $25 movie vouchers.

We used the Consolidated Criteria for Reporting Qualitative Research (COREQ) guideline statement to assist in the reporting of the study [28].

## Results

### Participants

A total of 204 participants participated in one of 24 FGs; participants were youth (*n* = 18) aged 16 to 17 years old, and young adults (*n* = 186) aged 19 to 29 years old. The study team conducted 13 FGs with participants that identified with the same sexual orientation or gender identity (3 gay, 3 bisexual, 3 trans, 2 lesbian, and 2 queer groups) and 11 FGs with individuals of varied gender identities and sexual orientations.

Participants had ethnic and cultural heritages that were very diverse, consistent with the population in Toronto and Ottawa; more than half(52%) identified as non-white. More than one-third of participants had completed high school (38%). Half of the participants reported that they rent or own their homes (50%) and 23% reported insecure housing. 84% of individuals were daily or occasional smokers. Additional characteristics are depicted in Table 2.

**Table 2:** Demographic and smoking characteristics of focus group participants (*N* = 204)

CHARACTERISTIC	NUMBER (%)	CHARACTERISTIC	NUMBER (%)
Age		Have you smoked 100 cigarettes?	
16-17	18 (8.8)	Yes	186 (91.2)
18-29	186 (91.2)	No	16 (7.8)
Total	204 (100.0)	Missing	2 (1.0)
Mean Age	23	Total	204 (100.0)
Gender		Ethnicity	
Female	85 (39.0)	Aboriginal	25 (10.4)
Male	58 (26.6)	Black/African/Caribbean	46 (19.1)
Trans Female	8 (3.7)	Central Asian	1 (0.4)
Trans Male	15 (6.9)	East/South East Asian	16 (6.6)
Two-Spirit	9 (4.1)	Latin America	12 (5.0)
Gender-Queer	32 (14.7)	Middle Eastern	7 (2.9)
Intersex	1 (0.5)	South Asian	11 (4.6)
Other^a^	10 (4.6)	White	115 (47.7)
Total^b^	218 (100.0)	Other	8 (3.3)
		Total^b^	241 (100.0)
Sexual orientation		Education	
Lesbian	27 (12.9)	Some high school (currently enrolled)	25 (12.3)
Gay	54 (25.8)	Some high school (not currently enrolled/not completed)	21 (10.3)
Bisexual	57 (27.4)	Completed high school with diploma	78 (38.2)
Queer	51 (24.5)	College degree^d^	35 (17.2)
Transgendered Heterosexual	5 (2.4)	University degree^d^	40 (19.6)
Pansexual	10 (4.8)	Graduate degree (Masters or PhD)	4 (2.0)
Other^c^	4 (1.9)	Missing	1 (0.5)
Total^b^	208 (100.0)	Total	204 (100.0)
How soon after waking do you smoke?		Housing	
<5 minutes	25 (12.3)	Living with parent	59 (25.2)
6-30 minutes	50 (24.5)	Rented or owned	118 (50.4)
31-60 minutes	31 (15.2)	Homeless	12 (5.1)
>60 minutes	64 (31.4)	Social Housing	17 (7.3)
I don’t smoke	17 (8.3)	Couch-Surfing	25 (10.7)
Missing	17 (8.3)	University/College Residence	3 (1.3)
Total	204 (100.0)	Total^b^	234 (100.0)
Currently smoke?		Years lived in Canada	
Daily	113 (55.4)	0-1 years	12 (5.9)
Occasionally	58 (28.4)	2-5 years	17 (8.3)
Recent quitter	30 (14.7)	6-10 years	12 (5.9)
Missing	3 (1.5)	Over 10 years	163 (79.9)
Total	204 (100.0)	Total	204 (100.0)
Intend to quit in the next 30 days		City	
Yes	53 (26.0)	Toronto	156 (76.5)
No	32 (15.7)	Ottawa	43 (21.1)
Don’t know	89 (43.6)	Other	2 (1.0)
N/A	13 (6.4)	Missing	3 (1.5)
Missing	17 (8.3)	Total	204 (100.0)
Total	204 (100.0)		

^a^Gender fluid, gender neutral or non-binary

^b^The total number reflects the number of responses; some participants selected more than one response

^c^Demisexual, fluid, asexual or attracted to cisgender females

^d^Those who said “some college” or “some university” were recoded into “college” or “university”

### Key elements of prevention and cessation interventions for LGBTQ+ YYA

The results identified from the exploration of the three intervention scenarios are presented here. Eight themes arose during discussions of each of the three intervention scenarios, and were expressed across each type of FG. The themes serve as key intervention elements and are considered to be essential to program development. Each of these themes is described below with examples from one or more intervention ideas.

1. Interventions should be LGBTQ+ specific

Interventions for LGBTQ+ YYA need to be culturally modified or tailored to the LGBTQ+ community. For example, for the GCC intervention participants expressed the desire to gather with those of the same gender identity or sexual orientation:

*If I was in a group with just trans people, I feel like there would be so many different things to do together and talk about together and support each other. I think it would be a great idea.*

[Trans group participant]

Participants also articulated the desire for LGBTQ+ SM campaigns to be tailored to LGBTQ+ audiences and believed that would be a positive attribute:

*“I think it would be interesting to be watching television or YouTube and see an ad that directly speaks to me as a member of the LGBTQ community and as someone who currently smokes. I would really relate to it and I think that even the general population seeing some of them would also bring awareness to the fact that it's an issue in the first place.”*

[Trans group participant]

LGBTQ+ specific also meant gathering in a LGBTQ+ safe-space and having an LGBTQ+ specific counsellor:

***“*** *You meet at a place where you already feel comfortable and you're with people who, hopefully, you feel a sense of community or shared identity with.”*

[Queer group participant]

With the MPA, participants specifically indicated liking this intervention because the scenario was designed as an LGBTQ+ specific quit smoking app:

*“Assuming that it’s customised to LGBTQ, that it would incorporate the kind of struggles that...we’ve lived through, it wouldn’t be any ... average … quit smoking kind of app.”*

[Mixed group participant]

Similar to GCC, participants liked the fact that the MPA can be tailored to a specific LGBTQ+ group and that they could connect with a LGBTQ+ specific counsellor:

*“I think it’s good to have it geared towards the LGBTQ because that would be more incentive to be, like, ‘oh, well, I can connect with other queer youth…or I can connect with a counsellor who is queer-friendly.’”*

[Bisexual group participant]

2. Interventions need to be accessible in terms of location, time and cost:

Participants described how interventions need to be accessible in terms of location, time, and cost. For example, participants indicated that a GCC intervention should not be located “*in the brinks of town*” [Mixed group participant]. Participants also suggested using Skype or an online virtual communication platform (e.g., chatroom) to access a GCC program, as another potential delivery option:

*“I like the virtual idea just because it seems like something that doesn’t seem like a lot of effort. I find that going somewhere can stress me out more and then I’ll smoke more, but if it [were] virtual I could sit at home and just hang out there. That would be cool.”*

[Mixed group participant]

Limited time was a related barrier to location since if distances were far, and individuals have multiple commitments, a lack of time affects participation in group programming. Related to the barrier of location is limited time:

*“My schedule is so packed that I wouldn’t be able to attend anything. If it was online, maybe.”*

[Bisexual group participant]

Participants talked about an intervention being accessible, in real-time when the need arises:

*“I don’t think, honestly, that it [GCC] would do anything for me. I think it’s not really geared toward convenience at all. So, when you’re sitting there thinking about trying to have a cigarette, it’s not like two more days and then I can go to this thing and talk about it. It’s happening right now. I want something more readily available and something that can be comfortably used in your home. Something a little more accessible than usual.”*

[Mixed group participant]

Accessibility is the key reason why the MPA was so appealing to YYA participants.

*“It’s always available. It’s always in your hand. Everywhere you go you have access to it unlike weekly group meetings. If you’re feeling like you need to smoke and you’re actively trying to quit, you’ve got that resource that you can refer to… give you inspiration or encouragement… instead of your willpower alone. .”*

[Mixed group participant]

Participants also emphasized the importance of building an online web-based version of the MPA as many LGBTQ+ YYA do not own a smart phone:

*“I feel like there would need to be a website equivalent… [for] people who don’t have access to smartphones but do have access to public libraries. A lot of smokers are LGBTQ and a lot of LGBTQ are in poverty and homeless. The people that you want to access [the program] might not be able to access the program.”*

[Lesbian group participant]

Similarly, the wide accessibility potential of SM campaigns, as well as the limited effort required to access such an intervention were key reasons why they were favoured by many participants:

“*I feel that social marketing doesn’t just reach the LGBTQ community, but it reaches everybody … within our generation. We have a lot of access through it and I think that it can touch not only youth but parents and institutions, and all these places where… they need to get this information.”*

[Mixed group participant].

Lastly, participants expressed that any intervention that is offered, whether it be an MPA or GCC program, should be free of charge to use or access:

*“The really important thing about this is that it would have to be free. You could not make… an app that you pay for because that would create a really high barrier for a lot of people.”*

[Queer group participant].

3. Interventions need to be inclusive, relatable and highlight diversity

Any cessation or prevention intervention for LGBTQ+ YYA needs to be inclusive and include individuals of varying sexual orientations, gender identities, ethnicities, abilities and body types. This was echoed by different respondents across different FGs regarding the *Perks of Not Smoking Campaign (C)* and the MPA:

“*I love the idea as long as it’s inclusive, which would be cool … Make sure it includes trans folk and people of colour… ”*

[Mixed group participant]

*“If you had real LGBTQ people, not actors but real people, then that would be a lot better. And then you can relate to them and think, that could be me.”*

[Mixed group participant]

Ensuring inclusivity and broader representation, even within the LGBTQ+ communities, was reiterated frequently and was a key suggestion provided for the social marketing campaigns:

*“I don’t want to see young gay males, and… same sex couples. I want to see people who don’t have representation. I want to see a black trans woman… or I want to see something different. People have been desensitized to these images, and I think that the correct way to shift your perspective in order to make that new, and something that people are interested in engaging in, is to change who you’re representing.”*

[Mixed group participant]

Inclusivity and relatability also refers to involving LGBTQ+ persons in the intervention development process to ensure real life experiences are portrayed accurately:

*“I like the idea [of] the social media campaign with the web page, Facebook page, YouTube videos, Twitter feed, as long as it was created by LGBTQ youth, I think that has potential.”*

[Trans group participant]

4. Interventions need to incorporate LGBTQ+ peer support and counselling services

An important element, for any intervention, is having a good support or buddy system. With the GCC intervention, this was expressed multiple times by participants across the FGs:

*“I think the idea of having a group is really good because it’s such a social thing, smoking…If it were a group of people that you grew closer with or saw regularly then it would make sense to support each other.”*

[Mixed group participant]

The MPA was recognized by many as a potential intervention that could provide ongoing peer support and counselling services and was a main reason why it was liked by LGBTQ+ YYA:

*“I like the idea of a peer support network and some sort of moderated chat room …and I do like getting access to counselling services because it’s so hard to get those resources, maybe there could even be a counsellor linked up in the app.”*

[Mixed group participant]

For the SM campaign scenarios, the need for support emerged as a theme. For example, the *General Population Campaign (A)*, which aims to educate about LGBTQ+ issues, was liked by participants because the goal of this scenario was to raise awareness and compassion which in turn, may trigger important conversations within family members. This is a form of support that is much needed by LGBTQ+ YYA:

*“I like the stark reality that’s been portrayed by those commercials that talk about what LGBTQ face because I think that [it] does raise awareness and compassion and maybe people will be kinder [and] maybe they will make someone [feel] better and make them less wanting to reach for a smoke.”*

[Mixed group participant].

Similarly, for the *Awareness and Fact Campaign (D)*, participants expressed liking this campaign because it acknowledges the need to support each other as a peer group:

“*I like strength; it’s something that’s really important and highlighting our strength as the LGBTQ community because we are warriors. We’ve been through a lot. We fight [things] that cis-hetero people don’t even have to think about and so I think focusing on our strength and showing that we can come together and overcome tobacco, that’s great.”*

[Mixed group participant].

5. Need to integrate interventions with other activities or topics: do not just focus on smoking;

Participants communicated the need to build prevention and/or cessation interventions that incorporate activities or discussion topics that are not related to smoking or quitting and/or combining quitting smoking with other activities. Many participants iterated this for the GCC intervention, for example:

*“It’s [smoking] not something I’d want to talk about, but I can imagine coming to a group where there was something else that we were doing together as a group. I don’t know, a cycling group so you’d talk about cycling, if there was something we were doing together while also quitting smoking I can imagine it being something that I’d be interested in doing.”*

[Mixed group participant]

This theme also emerged, especially with the *Perks of Not Smoking Campaign (C)*, as participants expressed liking this campaign because it focused on the physical benefits of not smoking:

*“You look at all these good things, look how fast you could run if you wanted, look how much breathing you can do. I would be much more responsive to that and I know that a lot of other people would be as well.”*

[Mixed group participant]

Highlighting positive, non-physical benefits is also important and beneficial:

“*I like it being positive, but also showing other activities – not like I can play basketball or anything like that – but that I can focus on having a family now because I’m not putting my child at risk.”*

[Mixed group participant]

Integrating the intervention with other activities or topic areas also came up with the MPA intervention. For example, integrating the MPA with a health focus was an emergent theme:

*“Another idea is making it a health focus thing because if it’s also encouraging you to go for a run or go to the gym then you are not going to want to smoke because you will be coughing and not being able to breathe, and that is a part of that healthy lifestyle thing so it could be more integrated.”*

[Mixed group participant]

Participants also suggested including personal, motivational health challenges for the MPA intervention:

*“I liked the idea about [the app] motivating you… [M]aybe you could do a daily task, like going for a bike ride or going for a walk, and each day it's like ‘day two, why don't you go [for] a 20 minutes walk around the block’.”*

[Mixed group participant]

6. Interventions and/or messages need to be positive, motivational, uplifting, and empowering

With any cessation or prevention intervention that is developed, participants liked and preferred the interventions that had positive, motivational, uplifting and/or empowering elements. Across all three intervention descriptions, this was a reoccurring and salient theme. For example, there were many comments about liking the *Perks of Not Smoking (C)* and the *Awareness and Facts* (D) SM campaigns because they were positive campaigns:

*“I like C, anything positive I think is a go. So, yes, I think showing what the benefits are of you not smoking and how you would feel if you weren’t a smoker would be more positive than having people with holes in their neck on your cigarette packs.”*

[Mixed group participant]

The desire for an intervention with positive elements was also illuminated with the MPA and GCC interventions. For example, with the MPA intervention, participants said that the MPA would need to be fun, positive, engaging and “cool.” Similarly, with the GCC scenario, participants talked about the importance of gathering in a positive space:

*“It actually sounds pretty dope to be in a positive space where there’s someone there… who has methods and stuff and having other support and making friends and being in a positive space, that seems pretty chill*.”

[Youth group participant].

7. Interventions need to provide concrete coping mechanisms

Many LGBTQ+ YYA expressed the need for an intervention to provide concrete coping mechanisms to deal with the quitting process with the types of coping mechanism varying across intervention type:

*“You can’t just tell someone to quit and then, what are they going to do?... So [giving] alternatives or different things [they can do] and if they’re receptive and they want to quit, then it could help them.”*

[Mixed group participant]

A GCC intervention, for example, should provide nicotine replacement therapy (NRT):

*“I can see this working but you’d have to add options for smoking cessation, like the patch, gum, or nicotine inhalers. Or incorporate harm reduction because you’re either addicted to the nicotine, the hand-to-mouth habit or the social aspect of it. So having avenues to help reduce the harms of those and being able to talk about the things that trigger you to smoke.”*

[Trans group participant]

Similarly, participants suggested that SM campaigns and the MPA could highlight alternatives to smoking and highlight ways to cope:

*“[When people say] quit drinking, or quit smoking, they don’t say how it leaves a hole in your routine. It’s like…when you were asking about the group [counselling], and people were saying it would be good if there were outings or...like, physical activity. There needs to be some kind of routine thing that can replace [smoking*].”

[Bisexual group participant]

*“…it [app] gives you ideas of what’s going to happen once you quit smoking, maybe something to occupy your time when you would normally go out for a smoke. So maybe something that would find local, free things to do or other ways to spend your money or your time.”*

[Mixed group participant]

“*Maybe [social marketing campaigns] were advertising positive ways to cope with your anxiety or your social anxiety whatever you’re coping with*.”

[Mixed group participant]

With the MPA, participants suggested including games as a distraction or coping mechanism:

*“If there was a bunch of games on the app that were there to distract you from smoking, [you could] go play five minutes of a quick game instead of smoking.”*

[Mixed group participant]

8. Integrate rewards, incentives into the interventions

Finally, the provision of rewards, and/or incentives was considered to be an important component of quit smoking interventions. For GCC and the MPA, for instance, participants suggested having a reward system with prizes and incentives or perks such as food, public transit tokens, contests with prizes (such as a cash prize or iPods), and/or a certificate for completion:

*“Having some type of reward for quitting after a month or something, it doesn't have to be monetary, but rewards might help with that mindset of ‘I quit and I'm being rewarded for it,’ like a [one]-month incentive, two-month, whatever interval of time.”*

[Trans group participant]

This theme also emerged with the *Perks of Not Smoking* (C) SM campaign which focused on the financial benefits or rewards of not smoking:

*“I like… the campaign that tells all the benefits of not smoking, especially the money one. Like, oh, you’ll have 1,000 extra dollars a year by quitting.”*

[Trans group participant]

## Discussion

The purpose of this study was to obtain, from the LGBTQ+ YYA firsthand, feedback on key design considerations for potential interventions that could be incorporated for this diverse group. This is particularly important given the dearth of published research evidence on the effectiveness of LGBTQ+ YYA quit smoking interventions [14]. The intervention research to-date targeting the overall LGBTQ+ population is dominated by group and individual cessation counselling with or without NRT as well as large SM campaigns [24]. This study also explored how a MPA and SM could be used given the emerging trend in using this type of technology to improve health status [29].

Similar to the findings of Berger and Mooney-Somers review of 19 smoking cessation programs for LGBT people [16], participants in this study highlighted that tobacco use interventions need to be LGBTQ+ specific. This means that any intervention that is developed should be tailored and customized for their specific community (e.g., trans or lesbian), rather than the LGBTQ+ community as a whole, or the general population. The need for specialized programs is consistent with what is known for other types of interventions for the LGBTQ+ population such as substance abuse treatment [20, 30]. Although more rigorous research is needed to determine whether tailored or culturally modified interventions outperform non-tailored interventions in this population [24], recent review evidence suggests that LGBTQ+ tailored interventions appear to be effective for both short-term and long-term smoking cessation [16]. Qualitative research with LGBTQ+ adults confirms that tobacco prevention and cessation interventions should be tailored specifically to the community [10, 31] and research on the tailoring of interventions to the needs of the general population has shown an overall positive effect on health behaviour change [32]. The vocalized need for LGBTQ+  specific, culturally modified programs is also consistent with experiential evidence – that is, what is purported by the practice community who deliver on-the-ground interventions to the LGBTQ+ community [33].

Accessibility was another dominant theme related to program structure preference. LGBTQ+ YYA expressed that an intervention needs to be easily and readily accessible in terms of location, time, and cost. While this theme may not be new or different for other populations of smokers wanting to quit [34], it is especially important for the LGBTQ+ YYA population as it is well documented that this group faces unique societal challenges such as increased rates of poverty and homelessness [35]. Accessing an intervention that is centrally located, does not require expensive means of transportation, and/or a lot of time or money to access is of paramount importance to ensure greatest uptake and use by these communities. In addition, interventions should be readily available to use upon demand in order to help cope with unique stressors (i.e., of coming out, stigma, and living in a heteronormative and cis-normative society).

Perhaps one of the most vocally expressed sentiments by LGBTQ+ YYA was the need for any intervention to be “real.” This means the intervention needs to depict diverse and real images of the LGBTQ+ YYA population, including various body shapes and people of various ethnicities and sexual orientation (i.e., not just gay men). They also need to provide an accurate depiction and address real issues related to the LGBTQ+ YYA community, and involve LGBTQ+ YYA in the development process. Portraying diversity within the LGBTQ+ communities is best achieved by involving the people who are most affected by the issue or problem at hand. Community involvement and co-creation in the development and implementation of evidence-based programs is well documented in the literature as an important component of effective interventions [36, 37]. For LGBTQ+ YYA, this key ingredient goes beyond program effectiveness; it speaks to the authenticity and genuineness of an intervention in helping this target group. The population is more vulnerable and at a higher risk to experience long term stresses related to their sexual orientation and/or gender identity [11]; so, any intervention that is not perceived to be genuine will contribute to the frustrations and challenges already faced.

The need to incorporate peer support and counselling services is not new to cessation interventions [38, 39]. For example, network analysis of the Framingham Heart Study cohort demonstrated that having a social contact during quitting smoking increased a smoker’s chances of quitting [40]. Further, a systematic narrative review of eight studies has suggested more promising results when peer-support is implemented as a smoking cessation method in economically and socially disadvantaged populations [41]. Participants from these FGs indicated a preference for LGBTQ+ specific peer support and counselling services. This relates closely with the desire for this population to have LGBTQ+ specific interventions and for interventions to be relatable and “real.” This population has culturally distinct and unique needs that demand specific attention and focus [36]. Recognizing and understanding needs among LGBTQ+ YYAs is one of the first steps in developing an intervention that will be successful in making a difference within this population group.

Participants liked the intervention scenarios that were positive, empowering and had motivational and/or uplifting messages. They disliked the scenarios that were upsetting or too negative. They also expressed disliking scare tactics. This, however, may be contrary to the evidence that exists with regards to the effectiveness of interventions such as mass media campaigns and graphic cigarette warning labels. For example, in a review of campaigns targeted at youth using a negative emotional tone, the negative campaigns had greater influence than campaigns with a positive or neutral tone [42]. Another review found that use of negative health effects messages increased knowledge, positive beliefs or motivation to quit as compared to how-to-quit messages, anti-industry messages and social norms themes [43]. In addition, research has shown that graphic health warnings on cigarette packages that elicit negative emotional reactions were associated with increased contemplation of health risks and promotion of cessation behaviour. These included fear-arousing health warnings, shocking images, personal testimonials, and depictions of human suffering or negative aesthetic effects [44]. Devlin and colleagues found that age was associated with the response to aversive imagery with younger respondents finding the short-term health and cosmetic effects more salient, while older smokers were more concerned with illness and premature ageing [45, 46]. Future research is needed to look at the effectiveness of negative imagery and messaging for LGBTQ+ YYA.

LGBTQ+ YYA expressed desire to add other non-smoking related activities and/or discussions as part of any intervention. Furthermore, this population group is looking for distractions, effective mechanisms to cope with the loss of smoking, access to NRT, and positive reinforcements in their cessation attempts. This is consistent with the findings of other studies addressing recommendations for the development of effective cessation interventions for LGBTQ+ YYA [11, 16, 36].

While three different intervention scenarios were discussed, participants suggested that all the components that were liked across the three scenarios could easily be integrated into one of the scenarios: the MPA with SM campaign intervention. There is a growing body of evidence supporting mobile phone-based interventions for smoking cessation although studies have primarily evaluated text messaging interventions for smoking cessation [47, 48]. In young persons (aged 18–29), smartphone ownership is as high as 86% and is closely reaching saturation [49]. The benefits to delivering a smoking cessation program through a MPA compared to traditional approaches include: low cost of the intervention, accessibility, portability, personalization, self-monitoring capabilities, location determining sensors, access to the internet, and ability to connect with social networking platforms [48, 50, 51]. However, despite smartphone ownership being almost ubiquitous among young persons, a smartphone intervention runs the risk of missing a small but important part of the community that is unable to afford a smartphone.

A MPA for cessation could be developed to be LGBTQ+ specific, involving and depicting the LGBTQ+ community, and providing LGBTQ+  specific supports. If developed in partnership with LGBTQ+ YYA, the MPA would likely be inclusive, relatable, and incorporate essential elements such as LGBTQ+ peer supports and counselling services. It could also incorporate other activities such as games and/or puzzles that distract from smoking and other concrete coping mechanisms, and integrate rewards and/or incentives as well as gamification throughout the quitting process [52]. The MPA itself could be accessible at all times for those who have a mobile phone, as it would be on their phone and accessible in real-time, when required. Lastly, the MPA could send encouraging and positive notifications about quit smoking progress. While numerous smoking cessation apps currently exist, to our knowledge, no LGBTQ+ specific smoking apps have been developed or tested for effectiveness [16]. The eight key elements that emerged from the analysis of FGs should be considered for incorporation into any prevention and/or cessation interventions designed for LGBTQ+ YYA. Future studies should incorporate a participatory action approach [53] to develop an intervention that incorporates all these essential elements.

### Limitations

This study has several limitations. We did not conduct analyses separately for each group so it is unknown if perceptions vary between LGBTQ+ populations. In addition, it is unknown if the findings from LGBTQ+ YYA in our sample will generalize to LGBTQ+ smokers in other communities and countries as the context for our sample is urban areas where services are typically available for those identifying as LGBTQ+ people.

During the FG sessions, we did not rotate the order of presentation of each of the three interventions. The order of presentation was consistently: 1) group cessation counselling, 2) social marketing, and 3) mobile phone app with social media. In order to avoid the tendency to respond to the most proximal when it came to reflecting on intervention preference, facilitators briefly re-counted each of the three interventions verbally. Participants were also able to re-read descriptions of each intervention handed out earlier during the session. Although 23 out of the 24 FGs consisted of 12 individuals or less, one FG included 17 participants, which may have negatively affected response quality for that particular FG. In an ideal scenario, findings would have been shared with all participants through a member checking process; this was conducted with a sub-set of participants who were successfully engaged. Despite the limitations, we identified an overall sample that was diverse in gender, ethnicity, sexuality, educational status, housing situation, and smoking status.

## Conclusions

LGBTQ+ YYA require tobacco prevention and cessation interventions that are uniquely designed for them. LGBTQ+ populations have smoking rates 2–3 times higher than the general population, face unique issues and challenges that the cisgender, straight community does not. Specifically, minority stress, stigma, marginalization, and tobacco industry marketing are among the key reasons that contribute to the significant health disparities experienced by the LGBTQ+ community [9, 54, 55].

This research contributes to and builds on the body of literature on smoking cessation interventions for LGBTQ+ populations and LGBTQ+ YYA in particular. Given the high need for cessation interventions for this community coupled with the lack of existing evidence-based options to fill this need, action is required to fill this considerable gap. With the input of FG participants, we identified the essential ingredients in building a cessation intervention that would be desirable to LGBTQ+ YYA. The mobile phone app is an intervention idea that can meet all the preferred criteria. Next steps include building an intervention with these essential ingredients using a participatory action approach that engages LGBTQ+ YYA in all phases of the development and implementation process. Both process and summative evaluations should also be conducted to learn about and understand the development process and implementation outcomes.

## Additional file

Additional file 1: LGBTQ+ Project: Focus Group Script and Questions. (DOCX 29 kb)

## Abbreviations

FG: focus group
GCC: Group cessation counselling
LGBTQ+: Lesbian, gay, bisexual, trans, queer, and other sexual minorities
MPA: Mobile phone application
SM: Social marketing
YYA: Youth and young adults
