# Supplementary material for: A qualitative study of tobacco interventions for LGBTQ+ youth and young adults: overarching themes and key learnings
Source: BMC Public Health. 2018 Jan 18;18:155. doi: 10.1186/s12889-018-5050-4 (PMC5774159; doi:10.1186/s12889-018-5050-4)
Supplement: Additional file 1: — LGBTQ+ Project: Focus Group Script and Questions. (DOCX 29 kb) [file 12889_2018_5050_MOESM1_ESM.docx]

**LGBTQ+ Project: Focus Group Script and Questions**

***Welcome and Introductions [3 minutes]***

Welcome everyone and thank you for coming. My name is … and I will be your facilitator for this focus group session We are working closely with some universities, Rainbow Health Ontario, and the Canadian Cancer Society to develop ways to help LGBTQ youth and young adults quit smoking or better yet, to not start smoking at all. We have ... here from Rainbow Health today. This is a really important area to address because LGBTQ youth and young adults are almost twice as likely to be smokers compared to non-trans and straight people of the same age and because smoking is extremely harmful to our health [if asked, 22% vs. 11% for youth as per the 2012 Atlantic Canada Drug Use Survey; 36% vs. 17% for young adults].

The purpose of this focus group is quite simple - we want your input and feedback. You know best what might work and what won’t work. Our discussions will have two parts. First, we want to learn a little more about what you think about smoking. Knowing this will help us with the second part of this focus group at which point we would like to get your input on three specific ideas for preventing smoking and encouraging quitting. I will be keeping us on track with the use of my [phone/watch] as a timer. This is to make sure we get through all the materials and we don't keep you for longer than we promised.

Before we begin, I just want to remind you of a few things. You may choose to not answer any question that you are asked. This discussion group is being audio-recorded to make sure everything is captured accurately, we also have a note-taker here. All information you provide is confidential although we may use some anonymous quotes when we share what we have learned. These are a few of the ground rules for this focus group: 1) please be respective of everyone’s perspective; 2) one person speaking at a time, please; 3) we have 1.5 hours allotted for this focus group and I will be keeping us on schedule 4) there are no right or wrong answers; we are looking for your opinion and feedback; We also please ask that you don’t share what other people say once this focus group is over. Your $50 will be handed out to you at the end of this session.

**Does anyone have any questions before we begin? [Note-taker – please write down any questions that are asked including initials of who asked] [5-10 minutes]**

Ok – I would like to start this session by hearing from each of you. Can everyone please introduce themselves and share what movie they last watched. When you introduce yourself, please use the name that you wrote down when you registered today and say your preferred gender pronoun (e.g., she/he/him/her). [1 minute per person max; 10 minutes max- facilitators, also please respond to this question so the group can warm up to you as well].

***Attitudes Regarding Tobacco Use Questions***

Great. Ok! Let’s get started. I want to hear how you feel or what you think about smoking. Remember, there are no right or wrong answers. We simply want to know what you think.

1. **How do you feel about smoking? [5 minutes]**

PROBE: What do you like about smoking? [For recent quitters] What did you like about smoking? What don’t you like about smoking? Why did you start smoking? When do you smoke the most?

1. **How do you feel about quitting? [5 minutes]**

PROBE: Is quitting important to you? [For recent quitters]: Was quitting important to you? [Have you ever tried quitting?] When you last tried to quit how did it go?

1. **If you were to quit smoking, what would help you quit?; [Recent quitters]: Was there anything that helped you quit? [5 minutes]**

PROBE: What made you think about quitting? What helped you? [Do not read out initially but if they struggle facilitators can give some of the following ideas: e.g. patches, gum, counseling, Quitline, friends, etc.]. What didn’t work for you?

***Intervention Ideas for Feedback [1 minute]***

Great! Thanks for sharing your experiences. Now, we’re going to switch gears and ask for your input on three different ideas that may help LGBTQ youth and young adults quit and stay smoke-free.

I’m going to hand out each option. We’re going to read it together and then we’re going to discuss it. We’ll do the same for the other two ideas. At the end, we’ll compare the three ideas and then discuss which one you liked best and least and why. Does anyone have any questions?

While I’m reading each option, please write down any questions or thoughts you have and we can discuss them after I’m done reading. Again, there are no right or wrong answers. We simply want to get your feedback.

**Idea #1: Group Cessation Sessions [15 minutes]**

This is the first option [facilitator hand out first option]. [Facilitator begins reading option 1].

Imagine that every week you could meet with other LGBTQ youth and young adults who want to quit smoking. A LGBTQ counsellor would run the meetings in a safe and accepting space. The counsellor would share trusted information on how to quit and stay smoke-free, but would leave time for group members to talk about personal experiences with smoking and quitting. Examples of information that might be shared include isolation, loneliness, body image, lifestyle changes, the need for positive support, stressors like the coming-out process, triggers and self-esteem. These groups could be a way to connect with others your age with similar experiences, and promote LGBTQ people supporting LGBTQ people to overcome smoking. The group would also encourage participants to buddy-up with other members so that during the week, people would be able to support each other to stay smoke-free. The group sessions would be 6-8 weeks and have 8-15 people.

**Questions:**

1. So, how do you feel about a group cessation program for people your own age who are also LGBTQ, who smoke, and who want to quit smoking?
2. Can you imagine yourself attending such a program to help you quit smoking? Why or why not?
3. What are some things that you like about a group program?
4. What are some things that you don’t like about it?

[Facilitator –ask participants to set scenario 1 aside]

**Idea #2: Social Marketing Campaign [20 minutes]**

This is the second idea that we will discuss [facilitator hand out second option]. [Facilitator begins reading option 2].

Here are four media campaign ideas that we would like your feedback on. I will read out all four first and then we’ll discuss what you think of them.

**This is social marketing campaign #2A [Anti-discriminatory campaign to educate the general population]**

Rather than a campaign that focuses on the LGBTQ community, maybe it’s time to educate the general population about some of the challenges faced by LGBTQ people. Many LGBTQ people experience homophobia, transphobia, heterosexism and are unfairly treated by society. What about a campaign that shows, in first person, some of these hardships? The advertisements would show how challenges such as family stress, peer rejection, victimization, and social anxiety can lead to smoking. For example, in one scenario a young gay male is sitting with his parents and telling them that he is gay; his parents are clearly upset. Another scenario shows a same sex couple going on a date and hearing derogatory comments being murmured and being stared at. After each scene, the individuals are shown reaching for cigarettes to help them cope.

**This is social marketing campaign #2B: [Exposing the tobacco industry]**

Did you know that tobacco companies zero-in on the LGBTQ community because they think members of the LGBTQ community are an easy target? In fact, one of the biggest tobacco companies created a plan called Project SCUM to manipulate LGBTQ people into buying their cigarettes[if asked: SCUM stands for Subculture Urban Marketing]. Big Tobacco uses manipulative strategies, like sponsoring LGBTQ events and putting glamourized LGBTQ imagery in their advertisements to make it seem like they are allies. The truth is that these tactics are designed to exploit the community.

**This is social marketing campaign #2C: [Highlighting the positive attributes of the non-smoking lifestyle]**

What do you think about a campaign that would show the immediate benefits of not smoking, and the freedom non-smokers feel because they’re smoke-free? For example, ads may show LGBTQ individuals being active. There could be ads that show two young men running, or a young woman lifting weights. The slogan might say “I can run fast and free” or “Nothing can stop me.” Other immediate perks of not smoking that might be part of this campaign could be having more money, sex appeal, and smelling good.

**And lastly, this is social marketing campaign #2D: [Awareness and education about disparities/fear campaign/resilience/empowerment]**

Did you know that for every straight non-trans smoker there are at least 3 LGBTQ smokers? Also, did you know that smoking-related illnesses and deaths are also much higher for LGBTQ people? Not many people do. What do you think of a campaign that would raise awareness about smoking in the LGBTQ community? This campaign may also feature ads that talk about the challenges that LGBTQ persons have overcome, including smoking. For example, an ad may say: “I overcame the victimization; and the coming out process; I’m not going to let tobacco take me down.”

**Questions:**

1. How do you feel about media campaigns that can help with encouraging quitting or not-smoking?
2. Of the 4 ideas above, which one did you like the most? Why?
3. Which one did you like the least? Why?
4. For the one that you liked the most, is there anything that you would change?

[Facilitator – ask participants to put the social media scenarios aside]

**Idea #3 Mobile Health Techniques [15 minutes]**

This is the third idea that we will talk about today [facilitator hand out third option]. [Facilitator begins reading option 3].

Do you own a smartphone? Ever play Candy Crush or use Instagram? What if there was an app designed specifically for LGBTQ youth and young adults that could help you quit smoking? For example, this quit smoking app would allow you to create an individualized quit plan where you can set a quit date, it would provide feedback on how you’re doing, record what triggers you to smoke, and give you tips on how to remain smoke-free, as well as links to counselling services. One of the advantages of the app would be access to a peer support network which would connect you to other LGBTQ peers who are also trying to quit or who have already stopped smoking.

The app would be part of a bigger social media campaign that would include a webpage, Facebook page, Youtube videos, and Twitter feed with access to more detailed educational resources about smoking and quitting, for example, nicotine replacement therapy, like gum or the patch. LGBTQ role models would promote the campaign.

**Questions**:

1. How do you feel about a smartphone app and social media campaign for people your own age that are also LGBTQ, who smoke, and who want to quit smoking?
2. If a smoke-free app was customized to LGBTQ youth and young adults would you use it? Why or why not?
3. What are some things that you like about a smartphone app and social media program?
4. What are some things that you don’t like about it?
5. Has anyone tried any other quit smoking apps? If yes, which ones? Did you find them useful?

***[Facilitator– please ask participants to take out all scenarios and have them in front of them]***

***Reflection on all three ideas [10 minutes]:***

Let’s step back and reflect on the three ideas that we just discussed [have participants look at 3 ideas in front of them and summarize all 3 ideas]. Thinking back, we have the group program, the social marketing campaigns, and the smartphone and social media campaign ideas.

**Questions:**

1. Of the 3 ideas, which option or options do you like the most? Why did you pick this option or options?
2. Do you think this option or options would help you quit or stay smoke-free?
3. Do you think this option or options would also help your peers not smoke?
4. Do you have any suggestions for improving any of the ideas that we discussed?
5. Do you relate to the options that we discussed?
6. Do you have any other ideas that we haven’t discussed that you would like to share?
7. [If social marketing campaign is the favoured campaign and if there is time – ask about the preferred medium of the delivery of the campaign –e.g., radio, print, etc.]

***Closing script***

This concludes our time together. I would like to thank you for your time. Please leave the scenarios on the table as we will be collecting them. On a piece of paper in front of you, can you please indicate how you found out about this focus group (poster, word of mouth, Facebook ad, Facebook page, Supporting Our Youth). Your thoughts and opinions are greatly appreciated.

Before you leave, please see …, who will give you instructions on receiving your $50.
